# Climate Change and Medications: Implications for Clinical Practice, Healthcare Sustainability, and Pharmacoepidemiology

**DOI:** 10.1002/pds.70437

**Published:** 2026-07-31

**Authors:** Soko Setoguchi, Sean Hennessy

**Affiliations:** ^1^ Center for Climate, Health, and Healthcare Rutgers Health and RWJBarnabas Health New Brunswick New Jersey USA; ^2^ University of Pennsylvania Perelman School of Medicine Philadelphia Pennsylvania USA

## Abstract

1. The climate‐medication relationship is bidirectional: climate‐amplified exposures such as extreme heat affect medication access, stability, and safety/effectiveness (downstream), while the use and disposal of medications add to healthcare's carbon footprint, thereby contributing to the very warming that intensifies those exposures (upstream).
2. Downstream: Extreme heat, extreme weather, and supply chain disruption are concrete threats to drug stability and access, from mail‐order degradation of insulin and epinephrine to the Hurricane Helene IV‐fluid shortage, and environmental exposures may alter the effects of common medications.
3. Upstream: Therapeutically equivalent options can differ substantially in carbon intensity; eliminating unnecessary prescribing and selecting lower‐carbon therapeutic equivalents are the levers most directly available to prescribers and pharmacists.
4. The evidence base is still immature: current drug‐heat warnings rest largely on mechanistic evidence and case reports, and advancing the field requires linking environmental exposure data to medication outcomes through case‐crossover and time‐series designs, where confounding by indication and polypharmacy represent central challenges.
5. Where lower‐carbon options are already guideline‐concordant, clinicians and pharmacists can act now through prescribing choices, patient education on heat precautions and proper medication storage and disposal, along with healthcare system preparedness. Broader change requires guideline revision, formulary reform, transparent industy data, and collective action through professional societies, including the International Society for Pharmacoepidemiology.

## 1 Introduction

**FIGURE 1:**
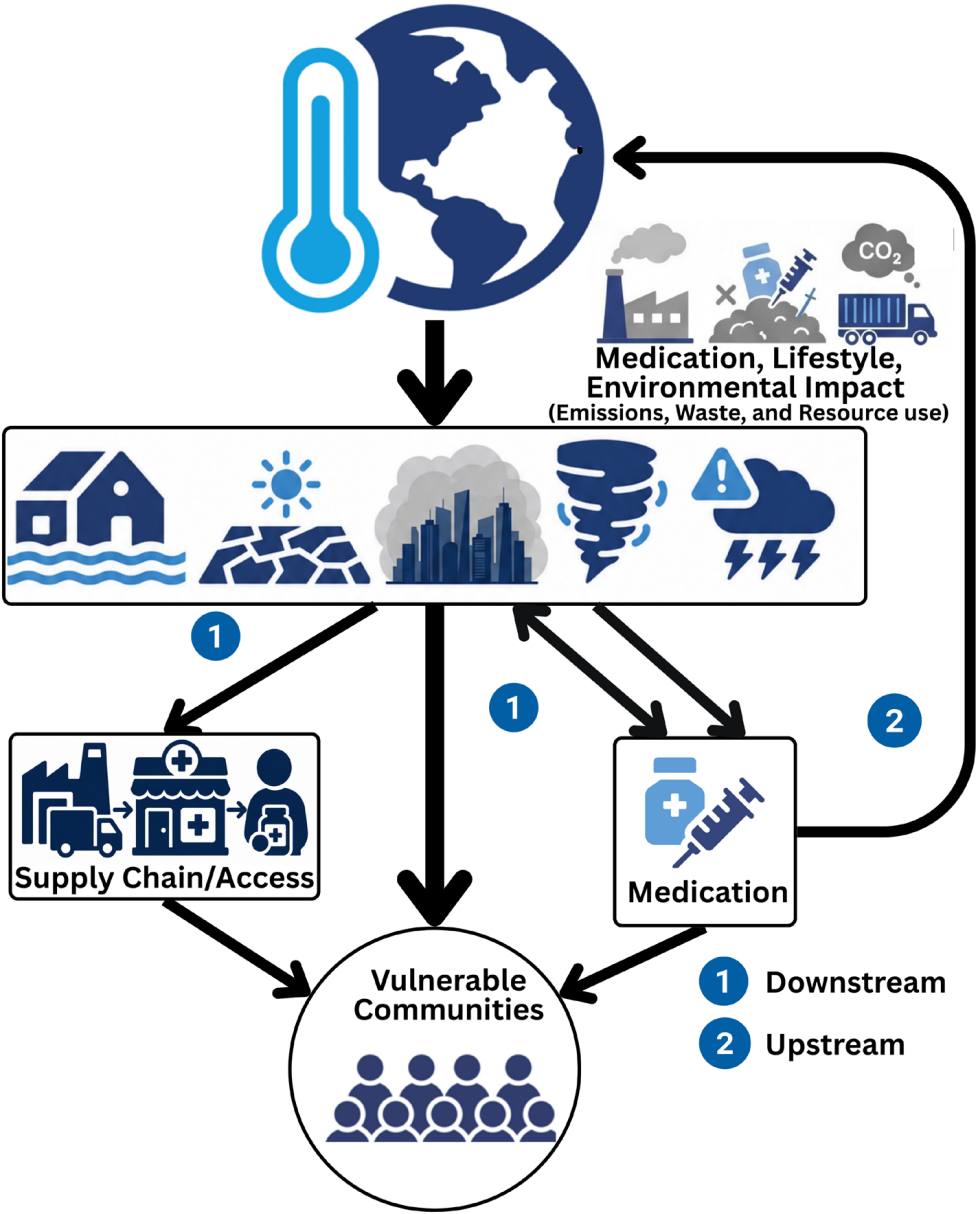
Climate‐Medication Interplays. Climate change (top) drives a range of climate‐related hazards, including extreme heat, drought, storms, flooding, and air pollution (center row). These hazards exert downstream impacts (arrows labeled ‘1’) on medications by disrupting supply chains and access, by interacting with treatment to affect drug stability, safety, and effectiveness, and by directly and indirectly harming health of vulnerable communities, who bear a disproportionate share of the burden. In the upstream direction (the looping arrow labeled ‘2’), medication use and treatment contribute to healthcare's carbon footprint and broader ecosystem impacts, further worsening climate change. The figure represents a conceptual framework rather than a quantitative model.

Climate change and medications each profoundly affect human health, yet their bidirectional relationship has received little attention (Figure 1). Climate‐driven exposures threaten drug stability, access, safety, and effectiveness of medications [1]. Meanwhile, prescribing choices and medication overuse contribute to healthcare's carbon footprint, estimated at 8.5% of national CO_2_ emissions [2]. Given that 60% of US adults and 85% of older adults use prescription drugs [3], this issue has broad relevance, with vulnerable populations bearing a disproportionate burden [4].

Climate change encompasses long‐term temperature shifts, altered precipitation, sea‐level rise, extreme weather events, and worsening air quality. This commentary focuses specifically on climate‐driven environmental exposures that are most directly relevant to medications, using a unified bidirectional framwork.

## 2 Climate Impacts on Medications (Downstream)

### 2.1 Temperature‐Related Drug Degradation

Millions of Americans receive prescription medications by mail, yet FDA guidance does not address the final stage of delivery to patients' homes. Non‐refrigerated drugs are recommended to be stored at 15°C–30°C, yet delivery trucks can reach 65°C, exposing mail‐order drugs outside recommended storage temperature for up to two‐thirds of transit time. Epinephrine, for example, loses much of its potency when exposed to 40°C–50°C, potentially jeopardizing emergency treatment [5]. Likewise, unopened insulin loses potency within months at 37°C. Power outages during extreme weather can therefore compromise insulin effectiveness during emergencies.

### 2.2 Climate Impacts on Supply Chains and Access

Climate disasters can disrupt medication supply chains nationwide. When Hurricane Helene flooded the Baxter facility, which produces approximately 60% of U.S. intravenous (IV) fluids, hospitals were forced to conserve scarce resources and shift to alternative therapies. This episode illustrates how one extreme‐weather event can jeopardize essential medication availability.

### 2.3 Joint Effects of Climate and Medications on Health

Many drug classes may increase heat‐related illness risk by affecting thermoregulation (e.g., cutaneous vasodilation, sweating, and cold‐seeking behaviors) or fluid and electrolyte balance. For example, diuretics and angiotensin‐converting enzyme inhibitors may blunt thirst sensation, and selective serotonin reuptake inhibitors and anticholinergics impair sweating [1]. Heat exposure can cause dehydration and renal injury that may reduce clearance of renally excreted medications. The CDC's clinical guidance for heat and medications lists these drug classes as potentially heat‐sensitizing [6], but the strength of evidence for these warnings varies considerably. Air pollution‐medication interactions have also been proposed, but only a handful of epidemiological studies have addressed this question [7].

## 3 Medications' Impacts on Climate Change (Upstream)

Pharmaceuticals account for up to 25% of NHS emissions in the United Kingdom and nearly two‐thirds of emissions in primary care. Overprescribing contributes to more than 1.5 million annual emergency department visits due to adverse drug events, a burden that carries both clinical harm and unnecessary environmental costs. In the United Kingdom, at least 10% of all prescription medications in primary care were judged unnecessary. Eliminating unnecessary prescribing and deprescribing [8] may be among the most effective strategies for clinicians to reduce the environmental burden of pharmaceuticals and improve patient outcomes, especially in populations with multiple chronic conditions and polypharmacy.

### 3.1 Differential Environmental Impacts of Therapeutically Equivalent Treatment Options

Although medication effectiveness and safety are widely studied, research on their environmental impacts remains limited. Where evidence exists, therapeutically equivalent options can differ substantially in their carbon footprints. Metered‐dose inhalers (MDIs) contain propellants with global warming potentials of 1430 to 3220 times greater than CO_2_, while dry powder and soft mist inhalers offer 10 to 37 times lower carbon intensity. Although many European countries have already reduced MDI use, MDIs constitute 88% of short‐acting beta agonists in the United States [9]. Similarly, when clinically appropriate, oral medications generally generate far less CO_2_ compared to their IV counterparts; oral antibiotics for bone and joint infections are already the guideline‐preferred route [10]; subcutaneous trastuzumab is non‐inferior to IV formulation [11]. Oral and subcutaneous administration also enables home‐based treatment, further reducing transport‐related emissions.

## 4 Evidence Gaps and Research Priorities

Although extreme environmental exposures have been studied under controlled conditions, little is known about the real‐world implications, such as high‐temperature exposure during mail delivery. Systematic assessments of supply chain vulnerabilities and impacts on use and access during extreme weather remain very limited.

Research remains limited on how changing environmental conditions influence drug effectiveness or safety (drug‐environment interactions). As a result, guidance for clinicians and patients on medication safety and adjustment during extreme weather events remains scarce and often rests on anecdotal evidence [6].

Limited product‐specific lifecycle assessment (LCA) and environmental impact data hinder clinicians' ability to recommend climate‐conscious prescribing choices when clinically appropriate. Comparative environmental impact studies of therapeutically equivalent treatment options are rare. Therefore, improving industry transparency and standardizing environmental reporting would help address the lack of drug‐specific environmental data.

These priorities differ in their research maturity, methods, and time horizons. Short‐term work can build on extensions of pharmacoepidemiologic methods by linking administrative claims to gridded environmental data using cohort, case‐crossover, and time‐series designs. However, methodological challenges arise both within and beyond pharmacoepidemiology, including (1) confounding by indication, (2) non‐identifiability due to collinearity among commonly co‐prescribed medications, (3) lack of standardized temperature exposure assessment (e.g., lag time, exposure windows [12]) and (4) measurement error from limited residential address [13] and mobility information [14].

Longer‐term priorities include LCA expertise and purpose‐built data, including prospective cohorts with detailed medication use and direct environmental monitoring, supply chain resilience data, community engagement, and impact assessments informed by climate projections.

## 5 Opportunities for Action

### 5.1 Climate‐Smart Prescribing and Medication Management

Individual clinician action is constrained by prevailing guidelines and formularies, which seldom consider sustainability; guideline bodies and payers therefore bear primary responsibility for embedding environmental criteria into prescribing standards. Other longer‐term changes include formulary reform, clinical decision support, and pharmacist‐led review programs. Nonetheless, where lower‐carbon options are already guideline‐concordant or formulary‐equivalent, clinicians can act now. Examples include favoring dry powder or soft mist inhalers over MDIs when appropriate, and selecting oral antibiotics or subcutaneous biologics where current evidence and patient preference support them. Clinicians can also review heat‐sensitizing medications in high‐risk patients ahead of summer and provide guidance on avoiding heat exposure. Pharmacists are particularly well‐positioned to counsel on storage during heat, flag heat‐sensitizing combinations at medication reconciliation, and lead deprescribing in older adults with polypharmacy. The CDC's clinical guidance on heat and medications [6] provides a practical starting point for identifying at‐risk drug classes and advising patients on heat precautions, though awareness of this resource among prescribers remains limited.

Deprescribing can simultaneously improve patient safety and reduce environmental impact. After appropriate assessment of each medication's ongoing benefit, clinicians can use resources such as www.deprescribing.org to safely reduce medication burden.

While a full account of overprescribing drivers is beyond the scope of this commentary, both clinician‐level factors (fear of malpractice, patient expectations, time constraints, habitual prescribing [15]) and system‐level factors (fragmented records, repeat‐prescribing processes, fee‐for‐service incentives) contribute. Established interventions exist, including clinician communication training, delayed‐prescribing strategies, decision support, and pharmacist‐led medication review.

### 5.2 Preparedness Integration

Responsibility for preparedness should be distributed among key stakeholders. Hospital systems and pharmacy networks should maintain backup power and emergency‐stock protocols; public health agencies should coordinate supply chain contingency planning and disaster communication; and payers, including CMS for Medicare and Medicaid populations, have a role in financing enhanced preparedness for high‐risk patients. Recent climate‐related disruptions have exposed existing supply chain vulnerabilities and justify prospective investment. In addition, patient‐specific risk stratification for drug unavailability must consider age, chronic disease burden, and social support. Higher‐risk patients require enhanced preparedness, including emergency supplies, backup storage options, and clear action plans covered by insurance. Patient education through healthcare providers should address proper storage during extreme weather, recognition of interaction symptoms, emergency action plans, and safe disposal methods.

### 5.3 Policy and Collective Advocacy

Because sustainability may not be a busy clinician's foremost priority, the most effective vehicle for advocacy is collective action through professional societies in related scientific fields, which can translate clinical and data expertise into policy influence, as demonstrated by the *Canadian Society of Internal Medicine Choosing Wisely Canada Recommendations* . Other stakeholders are also essential: manufacturers should be required to report lifecycle environmental impact data; pharmacy benefit managers who controls formulary design and tiering and insurers control formulary access for lower‐carbon alternatives; while regulators could incorporate environmental criteria into approval and labeling. System‐level incentives and institutional structures must carry the main burden of implementation.

## 6 Conclusions

The evidence base linking climate change and medications continues to mature, but the case for action is already clear across drug stability, supply chain resilience, environment‐medication interactions, and the environmental footprint of inappropriate prescribing and disposal. Several actions are warranted now: reducing overuse through curbing unnecessary prescribing, favoring lower‐carbon options where guideline‐concordant and aligned with patient preference, and educating clinicians and patients on heat precautions and proper medication storage, use, and disposal, along with strengthening health system preparedness for climate‐related care and supply disruptions. Acting now serves both current patients and future generations.

Achieving these benefits at scale will require more than individual effort. Responsibility is distributed across the system, from guideline bodies and payers to manufacturers, regulators, and professional societies such as the International Society for Pharmacoepidemiology (ISPE). The latter is well‐positioned to advance this agenda by generating evidence, advocating for transparent industry data and standardized environmental reporting, and integrating sustainability into research and practice. The time for climate‐smart pharmacoepidemiology is now.

## Funding

Dr. Setoguchi was supported by the National Institute of Child Health and Human Development, R01HD114056; National Institute on Aging, R01AG060232. The content is the sole responsibility of the authors and does not necessarily represent the official views of the NIH. Dr. Hennessy reports no funding for this work.

## Conflicts of Interest

Dr. Hennessy declares no conflicts of interest. Dr. Setoguchi reports receiving research funding from Pfizer Japan, and Daiichi Sankyo and serving as a consultant for Pfizer Japan, Merck Co. Inc., Regeneron, and Bristol Myers Squibb.

## Data Availability

Data sharing is not applicable to this article as no datasets were generated nor analyzed as part of the present study.
